# Diagnosis and Treatment of X-Linked Creatine Transporter Deficiency: Case Report and Literature Review

**DOI:** 10.3390/brainsci13101382

**Published:** 2023-09-28

**Authors:** Jiaqing Li, Sanqing Xu

**Affiliations:** Department of Pediatrics, Tongji Hospital, Tongji Medical College, Huazhong University of Science and Technology, Wuhan 430074, China; li.jiaqing@tjh.tjmu.edu.cn

**Keywords:** creatine transporter deficiency, developmental delay, MR spectroscopy, epilepsy, *SLC6A8*

## Abstract

(1) Background: X-linked creatine transporter deficiency (CTD) (OMIM 300036) is a rare group of inherited metabolic disorders characterized by global developmental delay/intellectual disability (GDD/ID), seizures, autistic behavior, and movement disorders. Pathogenic variants in the *SLC6A8* gene, located at Xq28, are causative of the disease, leading to impaired creatine transport into the brain. Supplementation with creatine and its precursors, glycine and arginine, has been attempted, yet the treatment efficacy remains controversial. (2) Methods: Here we report a de novo *SLC6A8* variant in a boy aged 3 years 9 months presenting with GDD, autistic behavior, and epilepsy. Elevated urinary creatine/creatinine ratio and diminished creatine peak on brain MR spectroscopy suggested the diagnosis of CTD. Genetic sequencing revealed a de novo hemizygous frameshift variant (NM_005629: c.1136_1137del, p. Glu379ValfsTer85). Creatine supplementation therapy was initiated after definitive diagnosis. Electroencephalography and MR spectroscopy were monitored during follow-up in concurrence with neuropsychological evaluations. The clinical phenotype and treatment response of CTD were summarized by systematic view of the literature. (3) Results: In silico analysis showed this variant to be deleterious, probably interfering with substrate binding and conformational changes during creatine transport. Creatine supplementation therapy led to seizure cessation and modest cognitive improvement after half-year’s treatment. (4) Conclusions: This case highlights the importance of MR spectroscopy and metabolic screening in males with GDD/ID, allowing for early diagnosis and therapeutic intervention. Mechanistic understanding and case-per-se analysis are required to enable precision treatment for the patients.

## 1. Introduction

Creatine is critical in ATP regeneration, therefore playing a crucial role in high-energy-demanding organs, including the brain, skeletal muscle, and kidney [1]. In addition, creatine may serve as an important neuromodulator in the brain [2,3,4]. It is synthesized from L-arginine, glycine, and methionine, mainly in the kidney and liver, and it is transported by sodium and chloride-dependent creatine transporter (CRTR) across the blood–brain barrier (BBB) into the brain [5].

X-linked creatine transporter deficiency (CTD) (OMIM 300036) is a recently identified inborn error of metabolism resulting from a deficiency of creatine transport into the brain. It has been reported to be the most common cerebral deficiency syndrome, accounting for approximately 2% of X-linked GDD/ID in males. Clinical symptoms include GDD/ID with speech and language delay, behavior abnormalities, seizures, and movement disorders [6]. Pathogenic variants in the *SLC6A8* gene, which encodes the creatine transporter, are associated with a spectrum of neurological symptoms.

The diagnosis of CTD is based on characteristic findings, including diminished creatine peak on MR spectroscopy, increased urinary creatine excretion, and *SLC6A8* sequencing. Despite extensive neurological examination, a definitive etiological diagnosis is often difficult to reach [6]. MR spectroscopy is a sensitive screening method to assess cerebral creatine content, but it is unavailable in many centers [7]. The urinary creatine test is becoming more available, and it can serve as a metabolic screening test in neurodevelopmental disorders [8,9]. In suspected CTD, the final diagnosis is confirmed by genetic sequencing of *SLC6A8* or a creatine uptake study in the cultured skin fibroblasts [10,11,12]. With the rapid development of next-generation sequencing, genetic tests for suspected CTD will become more common. Novel unclassified variants should be functionally characterized. Because it is not feasible to perform functional characterization for each identified variant, in silico analysis is emerging as a useful molecular diagnostic tool for prognosis prediction of the genotyping results. Early identification of developmental delays and appropriate management can positively influence the child’s developmental trajectory and outcome.

Currently, there are no approved treatment strategies for CTD. Creatine supplementation is administered as monotherapy or with creatine precursors to maximize creatine transport into the brain, yet the treatment efficacy remains controversial. The main barriers to biochemical and clinical improvement include the failure of creatine transport into the brain, as well as insufficient endogenous creatine synthesis and uptake [13,14,15,16].

In this study, we describe the clinical, biochemical, and molecular examination of a child suffering from CTD and his good response to oral creatine and glycine supplementation. The clinical and molecular findings were analyzed to provide mechanistic insight into the function of the SLC6A8 transporter. A systematic literature review was conducted to examine the effectiveness of the currently available treatment, which can be used to guide future intervention.

## 2. Materials and Methods

### 2.1. Patient and Data Collection

This study was conducted by the pediatric neurology department of Tongji Medical College, Huazhong University of Science and Technology. The patient was clinically evaluated and regularly monitored in our pediatric neurology ward. Peripheral blood was obtained from the proband and his parents after obtaining informed consent. This study protocol was approved by the Medical Ethics Committee of Tongji Medical College, Huazhong University of Science and Technology. Written informed consent was obtained from the patient’s guardians for the publication of this case report and the accompanying images.

### 2.2. Biochemical and Image Analysis

Creatine and guanidinoacetate concentration in blood and urine samples were measured by Gas Chromatography/Mass Spectrometry (GC/MS). Brain MRI and proton MR spectroscopy were acquired with a 1.5-Tesla system (HDMR; GE Healthcare, Chicago, IL, USA). The conventional MR imaging sequences included spin-echo T1-weighted images (TR/TE 500 ms/17 ms) and T2WI-fluid attenuated inversion recovery images (TR/TE 4000 ms/85 ms) in three orthogonal planes. Single-voxel point-resolved spectroscopy (PRESS) was performed with a voxel size of 15 to 25 mm and imaging parameters of TR/TE 1000 ms/144 ms. The Volume of Interest (VOI) was placed in the bilateral frontal, parietal, and occipital cortex and bilateral hippocampus and thalamus. Raw data were processed with software ADW 4.0 by GE. Further processing, including Gaussian apodization, 4-HZ zero-filling, Fourier transformation, water reference processing, frequency shift correction, and phase correction, was performed. Metabolic peak areas, including NAA, Cr, and Cho peaks, were determined using a curve fitting algorithm (NAA at 2.0 ppm, Cr at 3.0 ppm and Cho at 3.2 ppm).

### 2.3. Genetic Sequencing and In Silico Analysis

Whole-exome sequencing was performed on the Illumina HiSeq 2000/2500 platform (Illumina, San Diego, CA, USA) by Genokon Medical Laboratory (Xiamen, China). Sequencing reads were mapped to the human genome reference GRCh37/hg19 with a Burrows–Wheeler Aligner. The pathogenicity of the identified variation data was assessed using bioinformatics tools. The variants relevant to the patient’s phenotype were classified according to the American College of Medical Genetics and Genomics (ACMG) standards and guidelines. Multiple sequence alignment and analysis were performed and visualized in Jalview (https://www.jalview.org/, accessed on 8 July 2023). As the crystal protein structure of SLC6A8 is not yet solved, the *SLC6A8* variant (p. Glu379ValfsTer85) was mapped to the AlphaFold structure (AF-P48029-F1) to analyze the mutant structure effect. The three-dimensional structures were designed in PyMOL (https://pymol.org/2/, accessed on 8 July 2023) to visualize structural alterations.

### 2.4. Neuropsychological Assessment

The cognitive abilities of this child were assessed using Gesell Developmental Schedules (GDS) and the Wechsler Preschool and Primary Scale of Intelligence—Fourth Edition (WPPSI-IV test). His language ability was assessed with the Early Language Milestone Scale (ELS). The autism-like behavior was evaluated by the Autism Behavior Checklist (ABC) and the Childhood Autism Rating Scale (CARS).

### 2.5. Literature Search Method

All published case reports or studies on genetically confirmed CTD in peer-reviewed journals published in the English language were identified through a MEDLINE search (2001–June 2023) using the following MeSH terms: “creatine transport deficiency“ or “X-linked creatine transport deficiency“ or “SLC6A8 deficiency.” Studies and case reports were included when treatment with oral creatine supplementation and follow-up information were provided. Exclusion criteria: no intervention of creatine or creatine precursors, including no dosage or duration of treatment details; no follow-up information included. Full-text articles were assessed for eligibility. References cited by these papers were also evaluated to identify additional eligible publications.

## 3. Results

### 3.1. Case Presentation

This child was born out of a nonconsanguineous relationship with an uneventful prenatal and perinatal history (birth weight: 3.4 kg, birth length: 50 cm). Neurodevelopment in early infancy seemed normal, as he could raise his head at the age of 3 months. Simple focal seizures with secondary generalization were present from the age of 1 year and 9 months, presenting as eye deviation and stiffening of limbs. Most seizures were febrile with a short duration of less than one minute. He started to walk unaided at the age of 2 years. At the age of 3 years and 9 months, he still could not say “da-da” to dad or “ma-ma” to mom, and he was referred to the neuropediatric ward for further evaluation.

**Figure 1:**
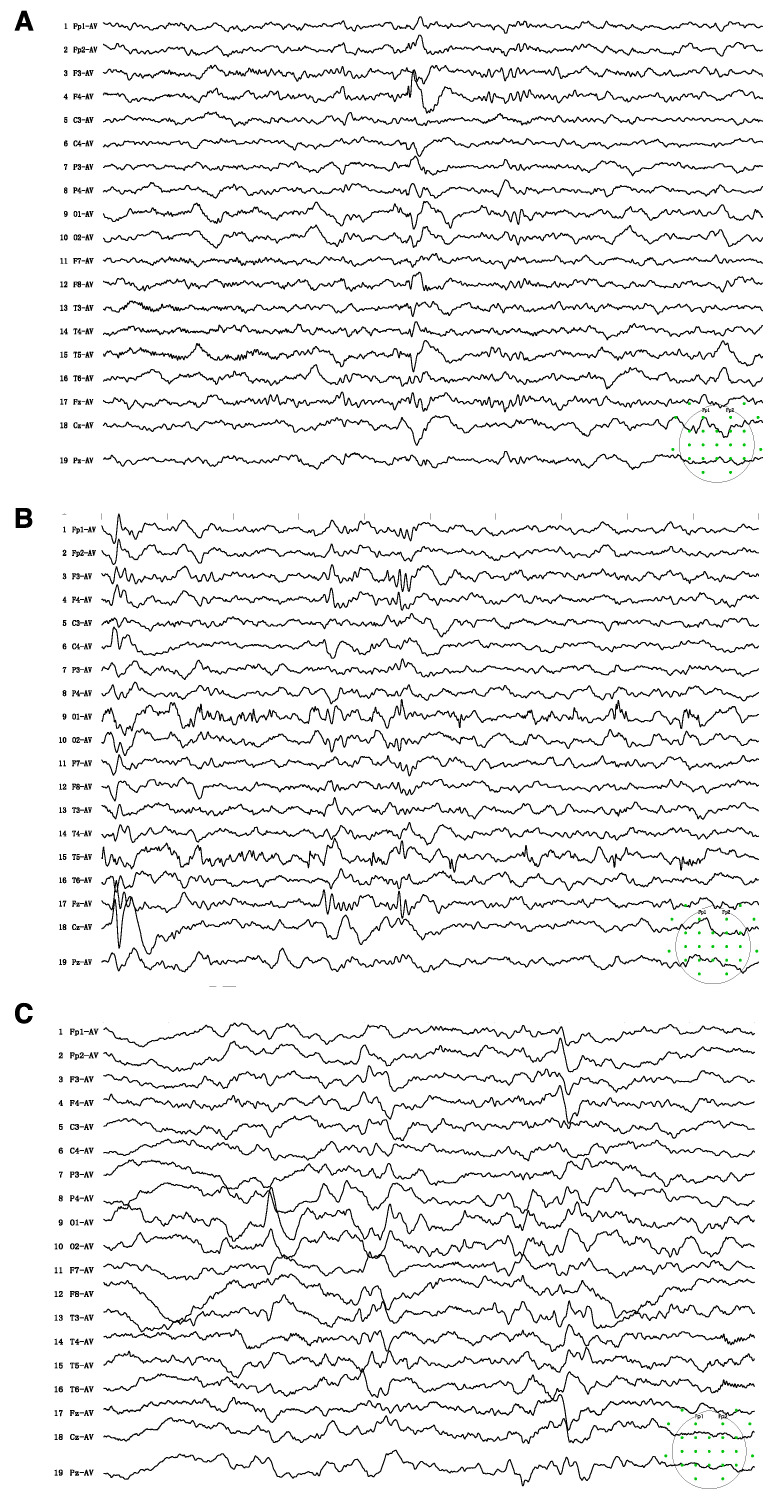
Electroencephalogram (EEG) on admission showed sharps and spike-wave discharges in the right frontal and anterior temporal regions (**A**) Subclinical seizures originating from left occipital and posterior temporal regions were recorded (**B**) Follow-up EEG after 6 months of treatment showed background slowing and runs of high-amplitude slow waves over the bilateral parieto-occipital and posterior temporal regions, particularly on the left side (**C**). The green spots indicated the montage of 10–20 system used for the present EEG recordings.

Upon examination, he was thinly built, with his height and weight below the third percentile of his age. The cranial nerve assessment was normal. He could walk with a broad-based gait, yet he had difficulty standing from a seated position. He could not cooperate for a formal muscle power assessment. The child showed autistic behavior, including attention deficit, poor eye contact, stereotypical movements, and poor response to his name. Therefore, the child was submitted to a comprehensive neuropsychological evaluation to assess his cognitive, speech, and social abilities. Neuropsychological assessment showed a global developmental delay, with severe speech and language delay. He had a full-scale IQ score of 42 in the WPPSI-IV test and poor performance across the five subdomains of gross motor, fine motor, language, personal–social responses, and adaptive behavior in the Gesell test (DQ = 47, 40, 22, 33, and 36, respectively). Electroencephalogram (EEG) recording revealed epileptic discharges at the right frontal and anterior temporal regions. Subclinical seizures originating from the left occipital and posterior temporal regions were recorded (Figure 1). The child was also subjected to cardiovascular evaluation, including electrocardiogram and echocardiogram, which revealed no abnormality.

**Figure 2:**
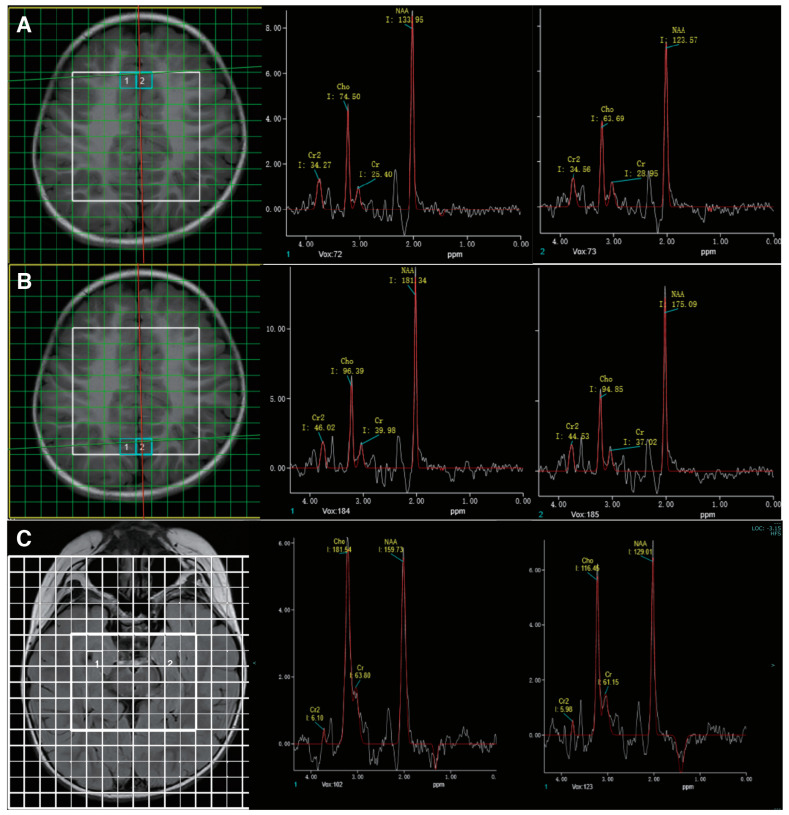
MR spectroscopy showed abnormally low creatine peaks in bilateral frontal (**A**) and occipital (**B**) regions. Follow-up studies six months after treatment showed no significant enhancement in the creatine peak (**C**).

The clinical presentation of the child suggested an inborn error of metabolism. Therefore, biochemical evaluation and MR spectroscopy, which are highly sensitive in detecting metabolic disorders of the central nervous system, were conducted. Biochemical analysis was performed with blood and urine samples, revealing an elevated urinary creatine excretion (3.46 mmol/mmol creatinine, normal: 0.005–1.07) and normal guanidinoacetate, which highly suggested the diagnosis of CTD. His MR imaging was unremarkable. MR spectroscopy showed diffusely diminished creatine peaks across the sampled brain regions (Figure 2).

### 3.2. In Silico Analysis of the Identified Variant

**Figure 3:**
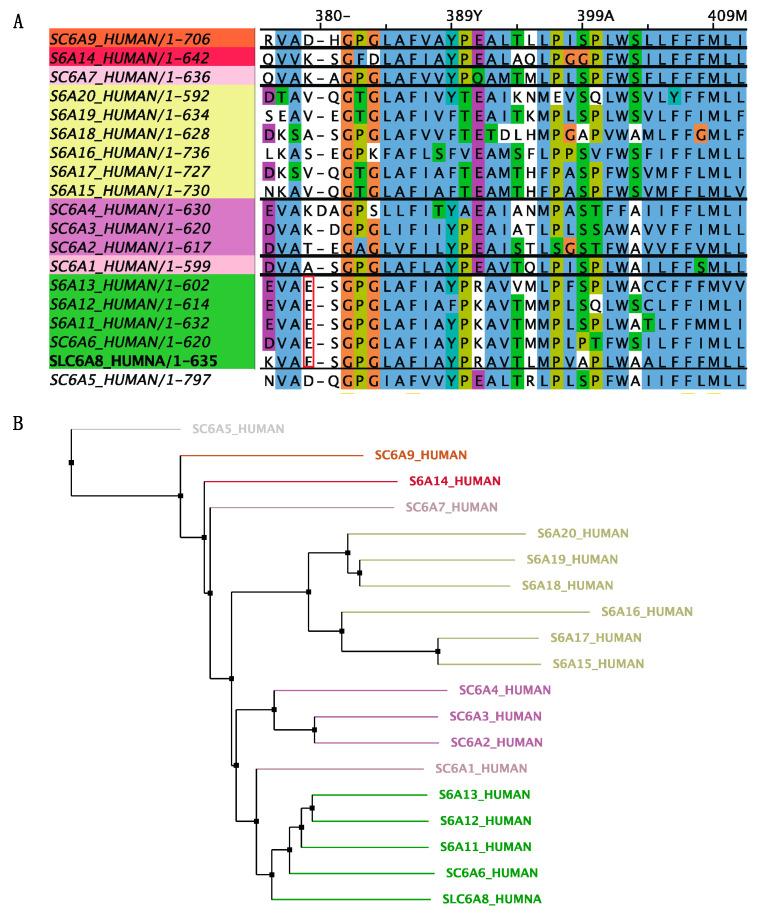
Multiple-sequence alignment with hierarchical clustering among the SLC6 superfamily. (**A**) The mutation site glutamate 379 residue is highly conserved between GABA transporter subgroups. All aligned sequences were saved from the NCBI database in FASTA format (https://www.ncbi.nlm.nih.gov/, accessed on 8 July 2023). Alignment was determined using the Jalview program. (**B**) Polygenetic tree of SLC6 neurotransmitter transporter family in Homo sapiens. The SLC6 family is divided into four groups, including the GABA transporter, amino acid transporter, monoamine transporter, and amino acid/orphan transporter. Polygenetic tree is built with neighbor jointing algorithms in Jalview program.

**Figure 4:**
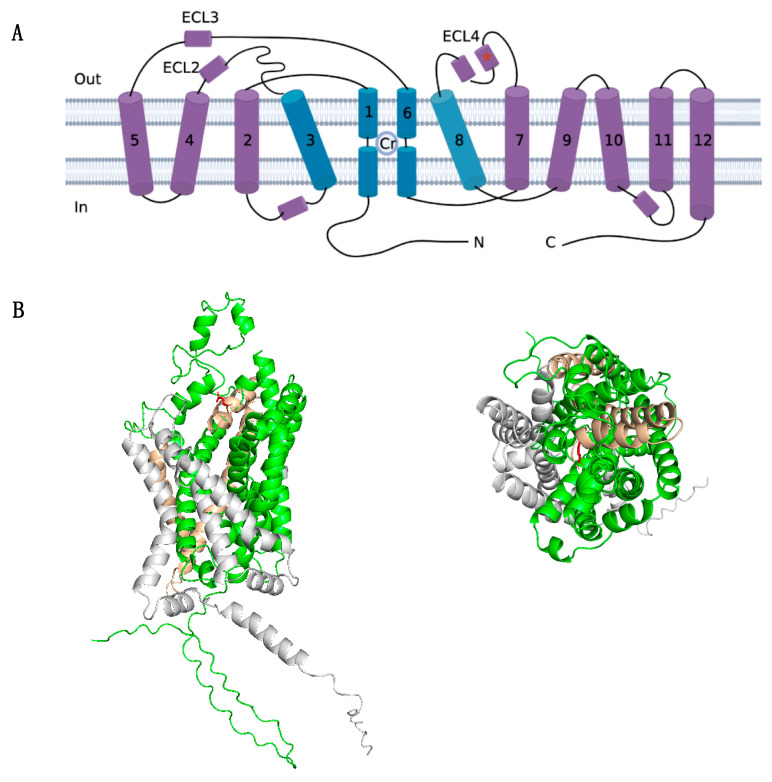
Structure analysis of the mutant SLC6A8 structure. (**A**) Schematic representation of the transmembrane topology of the creatine transporter. This transporter adopts the 12-transmembrane domain. The predicted binding site for creatine (Cr) is indicated. Transmembrane (TM) 1, 3, 6, and 8 (colored in blue) form the permeation pathway with the Cr molecule indicated. The mutation site glutamate 379 (in red asterisk) is located in extracellular loop 4 (ECL4). (**B**) Location of mutation site glutamate 379 presented in a solid ribbon model based on the AlphaFold structure (AF-P48029-F1) in PyMoL. The structure in green indicates the 1-379 amino acid of the original structure, the 85-amino-acid-long mutant structure is shown in yellow, and the truncated structure is shaded in grey. Mutation site residue is marked in red as a ball-and-stick model, which forms the external gate and covers the substrate binding site. Left: lateral view. Right: top–down view.

The diagnosis of CTD was confirmed by whole-exome sequencing, revealing a hemizygous frameshift variant in *SLC6A8* (NM_005629: exon7: c.1136_1137 del: p. Glu379ValfsTer 85). Sanger sequencing on parental samples revealed this variant occurred de novo. This variant was not found in the gnomAD database (Genome Aggregation Database, https://gnomad.broadinstitute.org/, accessed on 8 July 2023). ClinVar classifies this variant as likely pathogenic or pathogenic (variation ID: 2031214, https://preview.ncbi.nlm.nih.gov/clinvar/variation/2031214/, accessed on 8 July 2023). To exclude mitochondrial disease, parallel mitochondrial DNA sequencing was performed and showed no abnormality. The initial mutation site glutamate 379 residue is highly conserved between GABA transporter subgroups (Figure 3). And, the identified variant created a premature stop codon and resulted in a 464-amino-acids-long truncated protein with 1-379 of the original SLC6A8 protein (Figure 4). According to the ACMG guideline, this variant is classified as pathogenic (PSV1 + PS2 + PM2).

### 3.3. Treatment and Follow-Up

After the diagnosis of CTD, he was started on high-dose creatine monohydrate (400 mg/kg/d) and glycine (150 mg/kg/d) at the age of 3 years and 11 months. This patient also underwent speech, physical, and sensory integration therapy. Epilepsy gradually resolved after 3 months of therapy. After 6 months of treatment, a modest improvement in behavior was perceived by the family. The child had improved eye contact and social interaction. He had started to call “ma-ma”, but his verbal communication was limited to two to three words. He remained seizure-free upon regular follow-ups. After 6 months of treatment, the EEG showed background slowing and runs of high-amplitude slow waves over the occipital regions (Figure 1C). The brain MR spectroscopy did not show creatine peak enhancement (Figure 2C). The mother discontinued creatine supplementation therapy, leading to seizure recurrence in the following few weeks. At the follow-up visit, creatine therapy was resumed.

### 3.4. Systematic Literature Review of Creatine Treatment in CTD

A total of 82 studies were identified on MEDLINE. After initial screening, 15 studies met our criteria and were included in this study [17,18,19,20,21,22,23,24,25,26,27,28]. The 15 studies collectively describe 58 patients. A summary of their clinical details and treatment outcomes is provided in Supplemental Table S1. All male patients with CTD had GDD/ID with speech and language delay, with the severity ranging from no speech development (13%) to the ability to speak simple sentences (35%). About 86% of patients presented behavior disorders; the most prevalent were attention deficit hyperactivity disorder (41%) and autistic behavior (48%). Impulsive behavior, aggressive behavior, and self-injurious behavior were also reported. Seizures were present in 51% of patients with CTD, with various seizure types reported, including simple or complex partial seizures, generalized tonic–clonic seizures (GTCS), and myoclonic seizures. Other neurological clinical features were reported, including hypotonia, wide-based gait, spasticity, hearing loss, myopathic face, ptosis, and decreased muscle mass. Apart from neurological manifestations, symptoms from other systems were also reported, including gastrointestinal problems (e.g., diarrhea, constipation, feeding difficulties, failure to thrive) and cardiac symptoms (e.g., long QT syndrome, mild cardiomyopathy, premature ventricular contractions). Heterozygous pathogenic variants in the *SLC6A8* gene in females tend to have a milder phenotype than males depending on the X-chromosome inactivation pattern in different organs and tissues.

Treatment regimens varied among the fifty-nine cases: eleven patients received creatine–monohydrate supplementation; nine patients received L-arginine; thirty-four patients received a combination of creatine–monohydrate, L-arginine, and glycine; two patients received S-adenosyl methionine in conjunction with the triple therapy; two patients received triple therapy with creatine gluconate; and one patient in the presented case received creatine–monohydrate with glycine (Table 1). The treatment duration ranged from 2 months to 72 months. A total of 19 patients (32.2%) demonstrated response to treatment, manifested by either an increase in cerebral creatine or improved clinical parameters. Supplementation with the creatine precursor L-arginine and glycine seems a promising treatment in CTD. However, the efficacy of L-arginine supplementation alone is controversial. While clinical improvements were reported in four patients [20], in another study [17], five patients showed no improvement in neuropsychological assessment. The treatment with creatine monotherapy was discouraging [28,29,30,31]. Treatment with S-adenosylmethionine supplementation in conjunction with triple therapy seems promising, yet more case studies are required [23,26].

## 4. Discussion

Creatine deficiency syndromes are recently identified groups of inborn errors of creatine metabolism, including X-linked CTD (OMIM 300036) and two autosomal recessive defects of creatine synthesis (arginine–glycine amidinotransferase (AGAT, OMIM 612718) and guanidinoacetate methyltransferase (GAMT, OMIM 612736)). As creatine plays a fundamental role in maintaining high energy levels necessary for normal brain function and development [32], the brain is the primary organ affected by creatine deficiency; as such, the clinical hallmarks of CTD are mainly neurological symptoms, including GDD/ID, behavior abnormalities, and seizures [33]. In patients with CTD, the intellectual disability is pronounced with severe language delay, which was the main cause for seeking medical care. Development regression was also reported [24]. Behavior abnormalities mainly consist of autistic behavior, hyperactivity, attention deficit, and self-injurious behavior. Impaired brain energy metabolism and alteration in neuronal plasticity are the possible pathogeneses of neuropsychiatric disorders [34,35,36,37]. Seizures can be febrile-induced and comprise multiple types, including atonic, GTCS, partial, or complex partial seizures. While most seizures are drug responsive, severe epilepsy and status epilepticus were also reported [16,18]. There is growing evidence suggesting a strong involvement of brain energy depletion and mitochondrial dysfunction in the development of epilepsy [38]. Creatine supplementation was demonstrated to have potential anticonvulsant effects, yet the exact mechanism remains to be elucidated [39,40,41].

Given the heterogenous presentation, the diagnosis of CTD is often missed or delayed. Conventional MR imaging is important for routine evaluation in CTD patients, yet it shows no or mild structural and signal abnormalities, which are often not specific enough to suggest a definite diagnosis [7]. MR spectroscopy is highly sensitive and plays an essential role in the diagnostic evaluation of metabolic disorders of the central nervous system. In all patients with CTD, MR spectroscopy reveals severe reduction of creatine peaks in all brain regions [21]. Biochemical evaluation includes creatine, guanidinoacetate, and creatinine (Crn) measurements in urine and plasma. The elevated urinary creatine/creatinine ratio in CTD is probably due to reduced renal reabsorption. Creatine levels in plasma are normal. The interpretation of MR spectroscopic imaging and biochemical testing of urinary creatine/creatinine ratio together increase diagnostic accuracy, which can be further confirmed through genetic evaluation. In the present study, the metabolic profile of our patient fulfilled the biochemical criteria of SLC6A8 deficiency. The characteristic finding of diminished creatine peak on MR spectroscopy further confirmed the diagnosis of CTD.

Since its first report in 2001 [42], more than 80 pathogenic variants of SLC6A8 have been identified. The most common variant types are missense variants (38%), frameshift variants (23%), and 3-base pair deletions (19%). Other variants include splice variants, nonsense variants, and multi-exon deletions. All variants are listed in the LOVD3 database (https://databases.lovd.nl/shared/genes/SLC6A8, accessed on 8 July 2023). Genotype–phenotyp correlation analysis showed that missense variants with residual activity might be associated with milder phenotypes, while major changes (e.g., multi-exon deletion, frameshift, nonsense variants) would cause more severe phenotypes with failure to thrive, profound hypotonia, and movement disorder [7,24,43]. Structure studies enable more mechanistic insight into the likely impact of the variant [6,29]. In this study, genetic sequencing identified a de novo frameshift variant (c.1136_1137del, p. Glu379ValfsTer85) in the *SLC6A8* gene, creating a premature stop codon and resulting in a 464-amino-acids-long truncated protein. In silico analysis showed the identified variant is highly conserved in mammalian GABA transporters and located in extracellular loop 4 (ECL4), which is an important loop that covers the substrate binding site and is involved in conformational changes during creatine transport [44,45,46,47]. This massive structure truncation of critical parts is expected to trigger severe functional deficits, leading to global development delay, autistic behavior, and seizures. The same variant was reported in a child with severe ID and hypotonia [24], yet the clinical features and treatment outcome were not detailed.

Currently, there are no approved treatment strategies for CTD. Creatine and its precursors are administered as monotherapy or in combination to maximize creatine transport into the brain, yet the treatment efficiency remains controversial [1,6,22,24,48]. The challenge of treating CTD is mainly due to the low permeability of creatine across the BBB [13,49]. We systematically reviewed 59 patients with CRTR deficiency and their treatment outcomes (Table 1). A total of 19 patients (32.2%) demonstrated a response to treatment (creatine with its precursors 16/42 (38%), L-arginine 3/7 (42.8%), and creatine monotherapy 0/10 (0%)), manifested by either an increase in cerebral creatine or improved clinical parameters, including cognitive abilities, gross motor function, and epilepsy. And, there appears to be no correlation between the variant profiles and treatment responsiveness. This finding showed that combined creatine therapy with creatine precursors L-arginine and glycine seems to be a recommended treatment in CTD, while treatment with creatine monotherapy is probably ineffective. The efficacy of monotherapy with L-arginine supplementation remains to be determined due to limited cases. The treatment success is probably due to the enhanced endogenous creatine synthesis with creatine precursors (arginine and glycine), which can be transported into the brain by the cationic amino acid transporters CAT1 (SLC7A1, OMIM 104615) and CAT2 (SLC7A2, OMIM 601872) and glycine transporter 1 and 2 (SLC6A9, OMIM 601019 and SLC6A5, OMIM 604159) [50]. Future work should involve larger clinical trials with creatine as monotherapy and creatine with precursors. In addition, little is known regarding the optimal dosing for CTD, and the protocols used in the literature for creatine supplementation are heterogeneous [38,51]. Therefore, dose–response studies are required.

Currently, a lot of effort has been made to modify creatine molecules to enable transporter-independent creatine delivery into brain cells [52,53,54,55]. Lipophilic creatine analogs are considered an alternative treatment as they can cross BBB independent of CRTR, probably by simple transmembrane diffusion. Phosphocreatine-Mg-complex acetate creatine and creatinyl amino acids showed neuroprotective activity in vivo [56,57]. Cyclocreatine treatment showed creatine uptake and cognition improvement in *SLC6A8* KO mice [55,58]. Pharmacochaperoning is an additional promising new approach to rescue the folding deficit of CRTR [21,22,23]. *SLC6A8* missense variants produce misfolding proteins that often remain trapped in the endoplasmic reticulum. Pharmacological chaperones, such as 4-phenylbutyric acid (4-PBA), are small molecules that rescue the folding deficit of CRTR mutants, allowing its translocation to the appropriate cellular localization [15,54]. Over a third of folding-deficient variants were responsive to the functional rescue by 4-PBA, justifying the search for additional pharmacochaperones to restore misfolding of SLC6A8 transporters [15,54]. Gene therapies also appear as a promising avenue to treat CTD. The primary goal is to re-establish the expression of the functional SLC6A8 transporter in different brain cells. Among them, adeno-associated viruses have emerged as one of the safest and most used vectors for the delivery of therapeutic genes, with promising results in Parkinson’s disease and spinal muscular atrophy [59,60].

Several limitations of this study should be considered in the interpretation of the results, including (1) the risk of bias common to observational studies, such as selection bias, inadequate blinding, and selective outcome reporting; (2) inconsistency of effect, as demonstrated by high clinical or statistical heterogeneity; (3) imprecision due to the small sample size; and (4) publication bias. Another important consideration is the inevitable subjectivity in the interpretation of cognitive improvement, as formal testing was not performed in each study and the evaluation tools were different. Therefore, standardized neurodevelopment tests are required to determine clinical improvement.

## 5. Conclusions

CTD is an X-linked disease characterized by GDD/ID, autism, and epilepsy. Early diagnosis and treatment are crucial for the neurodevelopmental outcome. MR spectroscopy and metabolic/genetic analysis should be included in the diagnostic work-up for suspected CTD. We note that combined supplementation of creatine and its precursors is recommended for the treatment of CTD. In addition, novel effective treatment strategies, such as the design of CRTR-independent creatine analogs, pharmacochaperoning (for missense variants), and gene therapy through adeno-associated viruses, hold promise to improve the therapeutic outcome of CTD.

## Figures and Tables

**Table 1:** Summary of treatment trials with creatine precursors L-arginine and glycine.

References	Subjects		Treatment	Outcome
	N/Sex	Age		
Fons et al., 2008 [17]	4/M	9–16	Arg 400 mg/kg/d for 9 mo	No improvement in neuropsychological assessment; cerebral Cr not increased
Mercimek-Mahmutoglu et al., 2010 [18]	1/F	6	Cr 300 mg/kg/d, Arg 450 mg/kg/d + Gly 150 mg/kg/d for 28 mo	Resolution of intractable seizures; no other clinical effect; marginally increased cerebral Cr
Valayannopoulos et al., 2011 [19]	4/M, 2/F	2–16	Cr 400 mg/kg/d for 6 mo, then Cr + Arg 200 mg/kg/d + Gly 200 mg/kg/d for 12 mo, then Arg 200 mg/kg/d + Gly 200 mg/kg/d for 24 mo	No cognitive or psychiatric improvement, improvement in gross motor function in two males; cerebral Cr not increased
Chilosi et al., 2012 [20]	3/M	5–8.5	Arg 300 mg/kg/d for 24–36 mo	Seizure control; improvement in language and adaptive skills; creatine peak enhancement
van de Kamp et al., 2012 [21]	9/M	0–10	Cr 400 mg/kg/d + Arg 400 mg/kg/d + Gly 150 mg/kg/d for 48–72 mo	No measurable clinical improvement or deuteriation; cerebral Cr not increased
Dunbar et al., 2014 [22]	3/M	3–4	Cr 400 mg/kg/d, Arg 400 mg/kg/d + Gly 150 mg/kg/d for 28 mo for 5–33 mo	Improvement in neuropsychic functioning and speech; increased Cr/c by 1.2–4.3 times
Jaggumantri et al., 2015 * [23]	1/M	8	SAM (50 mg/kg/d) + Arg, Gly, and Cr for 3 mo	Significant improvement in speech/language skills; cerebral Cr not increased on MRS
Bruun et al., 2018 [24]	14/M, 3/F	1.2–7	Various regimens of combined Cr, Arg, and Gly therapy	None of the males showed either deterioration or improvements; two females showed improvements in the clinical severity score
Jangid et al., 2020 [25]	1/M	3	Cr 400 mg/kg/day + Gly 150 mg/kg/day for 12 mo, Arg 150 mg/kg/day for 3 mo	Modest motor and cognitive improvement
Yıldız et al., 2020 [26]	1/M	6	Cr 100 mg/kg/d, Arg 400 mg/kg/d, Gly 150 mg/kg/d, and SAM (up to 50 mg/kg/d) for 24 mo	Mild, subjective improvement in attention, expressive language, and behavior
Brugger et al., 2021 [27]	1/F	8.6	Gly 200 mg/kg/d, Arg 400 mg/kg/d, and Cr 400 mg/kg/d for 18 mo	Improvement in fine motor function, social behavior, and weight gain
Sun et al., 2023 [28]	2/M	0.7–2	Cr 400 mg/kg/d, Arg 300 mg/kg/d, Gly 150 mg/kg/d + Cr gluconate 400 mg/kg/d for 2–3 mo	Improvement in motor skills and cognition
this study	1/M	0.75	Cr 400 mg/kg/d + Gly 150 mg/kg/d for 6 mo	Improvement in cognitive function, speech skills, and social interaction

Abbreviation: F: female; M: male; mo: month; d: day; N: number; Cr: creatine; Arg: arginine; Gly: glycine; SAM: S-adenosylmethionine. * This patient received combined creatine therapy in Dunbar et al. [22], followed by S-adenosyl methionine treatment in Jaggumantri et al. [23].

## Data Availability

The data presented in this study are available on request from the corresponding author.

## Supplementary Materials

The following supporting information can be downloaded at: https://www.mdpi.com/article/10.3390/brainsci13101382/s1, Table S1: Summary of SLC6A8 variants with therapy and treatment outcomes.
