# Supplementary material for: Diagnosis and Treatment of X-Linked Creatine Transporter Deficiency: Case Report and Literature Review
Source: Brain Sci. 2023 Sep 28;13(10):1382. doi: 10.3390/brainsci13101382 (PMC10605349; doi:10.3390/brainsci13101382)
Supplement: Supplementary file 1 [file brainsci-13-01382-s001.zip › brainsci-2561128-supplementary.pdf]

Supplemental Table S1 Summary of SLC6A8 variants with therapy and treatment outcomes.

Literature	Sex	Age (yrs)	Genotype	Phenotype	treatment	response
**Bizzi 2002**	M	3.75	c.1221-1223 delTTC	severe ID, autistic behavior, seizure, motor delay	Cr/8 mo	ineffective
**Degrauw 2002**	M	6	c.1539C > T	severe ID, status epilepticus, hypotonia	Cr/4 mo	ineffective
**Fons 2008**	M	10	c.942-944 delCTT	severe ID, autistic behavior, intractable seizure	Arg/9 mo	ineffective
	M	16	c.1222-1223 delTTC	moderate ID, autistic behavior, seizure		ineffective
	M	13	c.878-879 delTC	severe ID, autistic behavior, seizure		ineffective
	M	9	c.1631C > T	mild ID, hypotonia		ineffective
**Mercimek-Mahmutoglu 2010**	F	6	c.1067G > T	moderate ID, ADHD, intractable seizure	Cr, Arg, Gly/28 mo	Resolution of intractable seizures, no other clinical effect
**Valayannopoulos 2011**	M	5	c.1519_1534del	mild psychomotor retardation and speech delay	Arg / 6 mo → Cr+Arg+Gly / 12 mo; → Arg+Gly 24 mo	no clinical improvement
	M	2	c.541T > C	seizure, autistic behavior, hypotonia		seizure free, improved muscle mass and gross motor function
	M	2	c.1221_1223delTTC	speech delay, HA		HA deteriorated
	M	2.5	c.1519_1542del	no speech, autistic behavior, seizure, muscular hypotonia		seizure free, improved gross motor skills, HA deteriorated
	F	16	c.291_292msAGGG	epilepsy, autistic behavior		epilepsy persisted; no cognitive function improved
	F	14	c.263-1G > C	epilepsy, autistic behavior (hallucination episodes)		reduced hallucination episodes
**Chilosi 2012**	M	8.5	c.1006-1008 del AAC	moderate ID, seizure	Arg/24-36 mo	seizure control, improvement in language and adaptive skills, creatine peak enhancement
	M	5.5	c.757G > C	mild ID, febrile seizure, hypotonia		
	M	5	c.1631C > T	Moderate ID, epilepsy, language deficit		

**Van de Kamp 2012**	M	10.3	c.1631C > T	moderate ID, ADHD, PDD NOS	varying regimes of combined treatment (Cr+Arg+Gly) over 4-6 yrs	Initial improvement on locomotor and social interactions but no lasting effect
	M	8.3	c.1631C > T	moderate ID, ADHD, PDD NOS, ODD		
	M	8	c.1495 + 5 G > C	moderate ID, ADHD		
	M	5.9	c.1495 + 5 G > C	moderate ID, ADHD, PDD		
	M	3.9	c.570-571 del	moderate ID, PDD NOS, ADHD		
	M	3.9	c.428-430 del	moderate ID, PDD NOS, ADHD		
	M	3.9	c.428-430 del	moderate ID, PDD NOS, ADHD		
	M	3	c.92delC	moderate ID, autistic behavior, seizure		
	M	0.75	c.92delC	GDD		
**Dunbar 2014**	M	3.2	c.1699T > C	severe ID, hyperactivity, autistic traits, hypotonia	triple therapy (Cr, Arg, Gly) for 5-33 mo	language progression
	M	4.7	c.859delC	mild ID, autism, seizure		improved neuropsychic functioning, muscle strength, communication, and interaction
	M	4.5	c.1222+1224del	moderate ID, hypotonia		improvement in gross motor skills, concentration, and speech
**Jaggumantri 2015 ***	M	8	c.859delC (reported by Dunbar 2014)	mild ID, autism, seizure	SAM+ Arg, Gly, Cr /3 mo	significant improvement in speech/language
**Bruun 2018**	M	7	c.1254 + 23_1274delinsCCTACA	severe ID, resistant seizures, ASD, ADHD, self-injurious behavior	Cr/24mo, Arg/24 mo, Gly/24 mo	better communication, less screaming
	M	4	c.1235_1248del14	mild ID	Cr/12mo, Arg/12 mo, Gly/12 mo	ineffective
	M	1	c.1421A > G	mild ID, febrile seizures	Cr/30mo, Arg/30 mo, Gly/12 mo	ineffective
	M	3	c.1667G > A	moderate ID, seizures, development regression, ADHD, aggressive behavior	Cr/13mo, Arg/52 mo, Gly/38 mo	ineffective
	M	8	c.1529 T > A	moderate ID, seizures, hypotonia, dysarthria	Cr/57 mo, Arg/13 mo, Gly/13 mo	improved physical activity and muscle strength

	M	1.8	c.262 + 1G > T	moderate ID, resistant seizures, autistic behavior, self-injurious behavior	Cr/27 mo, Arg/27 mo, Gly/27 mo	ineffective
	M	4	Known c.1428C > G	severe ID, seizures, autistic behavior, HA	Cr/12 mo	ineffective
	M	1.2	c.1136_1137del	severe ID, hypotonia	Cr/31 mo, Arg/23 mo, Gly/23 mo	improved muscle tone and power
	M	2	c.634G > T	moderate ID, hypotonia, wide-based gait, ASD, HA	Cr/48 mo, Arg/48 mo, Gly/48 mo	ineffective
	F	8	Known c.1067G > T	borderline ID, ADHD	Cr/48 mo, Arg/48 mo, Gly/48 mo	ineffective
	F	6	Known c.1067G > T	mild ID, refractotry seizures, ADHD	Cr/120 mo, Arg/108 mo, Gly/108 mo	seizure ameliorated
	M	6	IVS9 + 24 del24bp	severe ID, wide-based gait, ASD, aggressive behavior	Cr/24 mo, Arg/39 mo, Gly/39 mo	improvement in behavior and social interactions
	M	1.5	Known c.1684dupT	moderate ID, seizures, ADHD	Cr/46 mo, Arg/46 mo, Gly/46 mo	seizure responsive to phenobarbital
	F	2	Known c.456C > T	borderline ID, ADHD	Cr/7 mo, Arg/7 mo, Gly/7mo	ineffective
	M	7	Known c.321_323delCTT	severe ID, ataxia, ASD	Cr/30 mo, Arg/30 mo, Gly/30 mo	improvement in language comprehension, motor coordination, aggressive behavior
	M	4	Known c.1519_1543del	mild ID, ataxia, autistic behavior, ADHD	Cr/24mo	ineffective
	M	5	Known c.1A > G	severe ID, hypotonia, dysarthria, wide-based gait	Cr/72 mo, Arg/72 mo, Gly/72 mo	improved language and cooperation
**Jangid 2020**	M	3	c.507G>A	GDD, autism and epilepsy	triple therapy /12 mo	modest motor and cognitive improvement
**Yıldız 2020**	M	6	c.158dup	Moderate ID, speech delay, ASD, poor weight gain	tripple therapy+SAM /24 mo	mild, subjective improvement in attention, expressive language, and behavior
**Shi K 2021**	M	5	c.1222_1224del	Moderate ID, seizure, walking unsteadily	Cr /3 mo	improvement in muscle strength, no improvement in neurodevelopmental symptoms

**Brugger 2021**	F	8.6	c.1661C>T	Moderate ID, severe speech delay, hypotonia, ASD	triple therapy (Cr, Arg, Gly) /18 mo	improvement in fine motor function, social behavior, and weight gain
**Sun 2023**	M	8	Known c. 626_627delCT	severe ID, epilepsy	Cr / 6 mo	ineffective
	M	2	Known c.200G>A	severe ID, ADHD, laryngeal cartilage dysplasia, no language, epilepsy	Cr / 6 mo	ineffective
	M	7	Known c.778-2A>G	moderate ID, deficit in motor skills	Cr / 6 mo	ineffective
	M	2	Known c.1222_1224delTTC	severe ID, unsteady walking, inability to speak, epilepsy, constipation, ADD	Cr / 6 mo	ineffective
	M	1	Novel c.1767+1_1767+2insA	severe ID, repeated convulsions, ADHD, epilepsy	Cr / 4 mo	ineffective
	M	3	Known c.321_323 del	severe GDD, hypotonia, ADD, intractable epilepsy	Cr / 5 mo	ineffective
	M	2	Novel c.1496G>A	severe ID, open mouth, drooling, difficulty falling asleep, crying at night	Cr, Arg, Gly +Cr gluconate /2 mo	improvement in motor skills and cognition
	M	0.7	Novel c. 967G>C	GDD, little eye contact	Cr, Arg, Gly +Cr gluconate / 3 mo	improvement in motor skills and cognition
**this study**	M	3.75	known c.1136_1137	GDD, speech delay, autistic behavior, seizure	Cr+Gly/6 mo	improvement in cognitive function, speech, and social interaction

Abbreviation： F: female; M: male; yrs: years; mo: month; Cr: creatine; Arg: arginine; Gly: glycine; SAM: S-adenosylmethionine.
ID: intellectual disability; GDD: global developmental delay; ADHD: attention-deficit hyperactivity disorder; PDD NOS: pervasive developmental disorder not otherwise specified; ODD: oppositional defiant disorder; ASD: autism spectrum disorder; HA: hyperactivity; ADD: attention-deficit disorder.

* This patient received combined creatine therapy in Dunbar et al 2014, followed by S-adenosyl methionine treatment in Jaggumantri et al 2015.
